# Simultaneous Repression of *GLUCAN WATER DIKINASE 1* and *STARCH BRANCHING ENZYME 1* in Potato Tubers Leads to Starch With Increased Amylose and Novel Industrial Properties

**DOI:** 10.1002/biot.70051

**Published:** 2025-06-09

**Authors:** Muyiwa S. Adegbaju, Nina Gouws, Christell van der Vyver, Pedri Claassens, Jens Kossmann, Michaela Fischer‐Stettler, Samuel C. Zeeman, James R. Lloyd

**Affiliations:** ^1^ Institute for Plant Biotechnology Department of Genetics Stellenbosch University Stellenbosch South Africa; ^2^ Institute of Molecular Plant Biology ETH Zürich Zürich Switzerland

**Keywords:** Glucan water dikinase, Starch branching enzyme, Starch structure

## Abstract

This study examines how post‐transcriptional gene silencing of *STARCH BRANCHING ENZYME 1* (*SBE1*) and *GLUCAN WATER DIKINASE 1* (*GWD1*) affects the structure and properties of potato tuber starch. Silencing of either gene individually or simultaneously altered starch chemistry physical properties. Repression of *StGWD1* reduced phosphate content, while repression of *StSBE1* increased it. The phosphate content of starch isolated from plants where both genes were repressed was increased compared to *St* *GWD1* repressed lines, but lower than both the *SBE1* repressed lines and the untransformed control. Constituent chain lengths of starches from all lines were altered, and amylose content was increased in the *gwd1* and *sbe1/gwd1* double repressed lines, which also accumulated small numbers of lobed starch granules. Pasting properties were also affected, with starch from *StSBE1*‐repressed lines demonstrating increased peak and trough viscosities and *gwd1* lines showing decreased peak and trough viscosities, compared with the control. Peak and trough viscosities were lowest in the *sbe1/gwd1* repressed lines. We believe that these data demonstrate that alterations in starch phosphate influence the degree of branching within starch and offer a novel in planta strategy for optimizing the industrial properties of potato storage starch.

## Graphical Abstract and Lay Summary

Post‐transcriptional gene silencing was used to manufacture potato plants repressed in glucan water dikinase 1 and/or starch branching enzyme 1. This led to an altered starch structure, which affected its physical properties, meaning that the starch can be used in novel industrial processes.

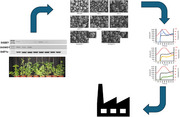

## 1 Introduction

The storage polyglucan starch is a major feedstock used by several industries, for example, as a thickener in some processed foods or as a binder during paper manufacture. Native starch must often be modified physically or chemically to change its properties before industrial usage, which adds cost. Starch is composed of two polymer fractions, amylose and amylopectin, which both contain α‐1,4 glucan chains linked together by α‐1,6 branchpoints [1, 2]. The main structural differences between these two polymers comes from their respective sizes and degree of branching. Amylopectin is larger and more highly branched than amylose and also contains a small amount of covalently bound phosphate [3, 4]. The structure of starch can affect its physicochemical properties, with the relative amounts of amylose and amylopectin, the degree of branching, granule size and morphology, and the amount of phosphate all contributing toward this [1, 5]. One aim of plant biotechnology has been to identify enzymes involved in starch metabolism and alter their activities in planta to change starch structure, leading to improved physicochemical properties for industrial uses.

Two classes of starch metabolic enzymes are glucan, water dikinase (GWD), which introduces covalently bound phosphate [6, 7], and starch branching enzyme (SBE), which forms α‐1,6 branchpoints [8, 9]. Starch phosphate is found attached to either the C6 or C3 positions of glucosyl residues within amylopectin [10] and is introduced by GWD1 [6, 11, 12] and GWD3 [13, 14, 15], which act both during starch biosynthesis and degradation. Phosphate is incorporated at the 6‐position by GWD1, while GWD3 phosphorylates at the 3‐position, acting only after the glucan has been pre‐phosphorylated by GWD1 [13, 14, 16]. GWD1 binds to starch granules [16], can penetrate into the surface [17], and preferentially phosphorylates α‐1,4 chains with a high degree of polymerization (DP), that is over 30 glucose moieties in length [7]. Transgenic and mutant potato plants have been produced where StGWD1 amounts were reduced, and starch from these plants contains greatly reduced amounts of covalently bound phosphate alongside increased amounts of apparent amylose [11, 12, 18, 19].

*SBE* genes can be divided into three clades [20], and members of two of these clades (*StSBE1* and *StSBE2*) have been studied in transgenic [21, 22, 23, 24, 25, 26] and mutant [27, 28] potato plants. Although StSBE1 contributes the majority of SBE activity in tubers, transgenic plants where its activity is greatly repressed still synthesize starch with no change in amylose content, but with significantly increased starch phosphate [21]. When StSBE2 is repressed, the amount of starch phosphate is also increased, and the branching of starch decreased [22]. Post‐transcriptional silencing of both isoforms simultaneously leads to the formation of starch containing both higher levels of amylose and starch phosphate than that found in either of the single repression lines [23, 24, 25, 26]. Interestingly, recent data suggest that in *Arabidopsis thaliana*, the third clade contains proteins neofunctionalized to a role in plastid gene expression and have lost branching enzyme activity [29], and a gene from that clade is also found in the potato genome [27, 30]. StSBE1 and StSBE2 are, however, responsible for all SBE activity in potato tubers, as when both were mutated, tuber starch containing only α‐1,4 chains was found [27].

The increase in amylose in *StGWD1* repressed lines [12, 19] and the increased phosphate in *StSBE* repressed lines [21–23, 25] indicate a relationship between starch phosphate and starch branching. Branching enzymes can utilize phosphorylated glucans as substrates [31], the phosphate introduced by GWD1 is found close to branch points [17, 32], and branching enzymes show differential affinity to phosphorylated and non‐phosphorylated α‐1,4‐glucans [33]. We hypothesized that this indicates a functional relationship between starch phosphate and branching, which might be demonstrated by simultaneously repressing both starch phosphorylation and branching. To this end, we repressed *StSBE1* and *StGWD1* either individually or together in potato to examine the effects on starch structure and physicochemical properties.

## 2 Materials and Methods

### 2.1 Plasmid Construction

RNAi plant transformation vectors, designed to repress *StGWD1* and/or *StSBE1*, were constructed by the following method. *S. tuberosum* RNA was extracted using the Qiagen RNAeasy Plant Mini Kit and stored at −80°C until further use. cDNA was produced using the Fermentas cDNA first strand kit and was used as a template in PCR. Fragments of 300 bp for the *StGWD1* and *StBE1* were amplified using primers shown in Table 1. Amplicons were purified using a PCR‐purification kit and ligated into pGem‐T‐easy (Fermentas). Gene fragments were restricted out of the pGem‐T‐easy vector using BamH1 and Sac1 for *StGWD1* and Pst1 and EcoR1 for *StBE1*. These fragments were then ligated either alone or together into the same site in pBK‐CMV (Agilent). The gene fragments in pBK‐CMV were amplified using primers (Table 1) that contain either *att*B1 or *att*B2 recombination sites and that bind to the T7 and T3 sites with pBK‐CMV. The amplicons were recombined into pHellsgate 2 [34] using BP Clonase (Invitrogen).

**TABLE 1:** PCR primers used for the amplification of gene fragments for the RNAi constructs and semi‐quantitative RT‐PCR.

Name	Primer sequence
Construct preparation	
GWD FWD	5’‐AT**GGATCC** TGGTGCTTCCATACAGGACA‐3’
GWD REV	5’‐AT**GAGCTC** TTCAGGTGCTTTTCCACCTT‐3’
BE1 FWD	5’‐AT**CTGCAG** CAGCTCTGAGCCACGTGTTAA‐3’
BE1 REV	5’‐AT**GAATTC** TGGCCAATATCAAAGCCATT‐3’
T7 Att	5’‐GGG**GACAAGTTTGTACAAAAAAGC** AGGCTGTAATACGACTCACTATAGGGC‐3’
T3 Att	5’‐ GGG**GACCACTTTGTACAAGAAAGC** TGGGTAATTAACCTCACTAAAGGG‐3’
Semi quantitative RT‐PCR	
SqEF‐1α FWD	5’‐ACTCCCCGGTGACAATGTTG‐3’
SqEF‐1α REV	5’‐TGGTCACTTTGGCACCAGTT‐3’
BE1 FWD	5’‐CCGAGCCCCACGAATCTATG‐3’
BE1 REV	5’‐ACAGCCTGCCTATCCCACAAC‐3’
BE2 FWD	5’‐CCGTTCAAGATGGGGGTGTT‐3’
BE2 REV	5’‐GGTGTTGTTCAGCCCTAGGG‐3’
GWD1 FWD	5’‐AGGTGGGAGAGGAAGGGAAA
GWD1 REV	5’‐TGTACTGCAGGACTGGAAGG‐3’

*Note: Att* and restriction enzyme sites are bold. Annealing sites are underlined.

### 2.2 Plant Transformation

Explant material was sourced from micro‐propagated *Solanum tuberosum* (cv. Désirée) plants grown on sterile Murashige and Skoog (MS) media (4.32 g L^−1^ MS with vitamins, 20 g L^−1^ sucrose, 0.5 g L^−1^ casein, 2.23 g L^−1^ Gelrite; Duchefa Biochemie). *Agrobacterium tumefaciens* strain GV2260 was transformed with the silencing vectors by electroporation. Transformed cells were selected at 28°C on lysogeny broth (10 g L^−1^ peptone, 5 g L^−1^ yeast extract, 10 g L^−1^ NaCl, 15 g L^−1^ Agar; Glentham Life Sciences) plates containing 50 µg mL^−1^ kanamycin, 50 µg mL^−1^ rifampicin, and 50 µg mL^−1^ spectinomycin for 48 h. Plant transformation was performed according to a previously published method [35].

### 2.3 Cultivation of Transgenic Plants

Selected transgenic potato lines were grown for 2 months on sterile MS media in tissue culture pots under a 16 h/8 h day/night cycle. Two‐month‐old transgenic plants were then potted in a soil mixture (1.5 parts potting soil and 1 part sand) and grown in ambient greenhouse conditions.

### 2.4 RNA Isolation and Expression Analysis

Plant tissue was homogenized by grinding the sample into powder with a mortar and pestle after the addition of liquid nitrogen and used for RNA isolation using Maxwell 16 LEV plant RNA kits and a Maxwell 16 MDx AS3000 (Promega) machine following the manufacturer's instructions. One hundred nanograms of RNA were converted to cDNA using the RevertAid First Strand cDNA Synthesis kit (Thermo Scientific) according to the manufacturer's instructions. Primers (Table 1) for semi‐quantitative PCR (sq‐PCR) were designed against potato cDNA sequences for the respective enzymes using Primer3 [36]. *Elongation Factor‐1α* (NCBI accession number AJ536671.1) was used as a housekeeping gene [37].

### 2.5 Immunoblots

Ten milligrams of potato tuber samples were homogenized in 200 µL of ice‐cold protein extraction buffer (100 mM MOPS pH 7.0; 2 mM EDTA; 1 mM DTT; 10% [v/v] ethanediol), centrifuged at 13,000 × *g*, and the supernatant containing crude soluble protein was transferred to a fresh microcentrifuge tube. Total protein was determined using a commercially available kit (Thermo Scientific). Twenty micrograms of total protein were denatured by incubating at 95°C for 5 min in 2% (w/v) SDS, 10% (v/v) glycerol, and 60 mM Tris‐HCL (pH 6.8) prior to separation on 8% (v/v) SDS‐PAGE at 100 V for 90 min. The gel was blotted onto nitrocellulose using a semi‐dry blotting system (BioRad), and the membrane was probed with anti‐GWD1 antibody [11] diluted 1:500 in PBS‐T. After washing, the membrane was incubated with anti‐rabbit IgG bound to alkaline phosphatase secondary antibody (Sigma; diluted 1:7500 in PBS‐T) for 2 h. The presence of GWD1 protein was visualised by incubating it in BCIP/NBT (5‐bromo‐4‐chloro‐3‐indolyl phosphate/nitro blue tetrazolium) solution (Sigma*FAST*, Sigma).

### 2.6 Starch and Soluble Sugar Determination

Starch and soluble sugars were determined enzymatically according to a previously published method [38].

### 2.7 Purification and Analysis of Starch

Starch was isolated from potato tubers by grinding in buffer, filtering through Miracloth and allowing granules to settle by gravity according to a previously published method [39]. An enzymatic assay [40] was used to determine the glucose‐6‐phosphate content of the purified starch by hydrolyzing purified starch by heating in 0.7 M HCl for 4 h at 95°C, and then determining glucose by measuring the increase in absorption at 340 nm after the addtion of ATP, NAD (both from Megazyme), hexokinase (from yeast), and glucose‐6‐phosphate dehydrogenase (from *Leuconostoc mesenteroides;* both enzymes are from Megazyme) for glucose or glucose 6‐phosphate dehydrogenase and NAD alone for glucose 6‐phosphate. The apparent amylose content of purified starch was determined by two methods. Firstly, using an iodine binding method where Lugol's reagent is mixed with starch leached from purified granules by 45% (v/v) perchloric acid and absorbance measured at both 618 and 550 nm. Amylose was calculated using the equation described previously [41]. Secondly, using a commercial kit based on the precipitation of amylopectin by the lectin concanavalin A (Megazyme). Swelling power was determined by the method of Howard et al. [42], where purified starch was heated in water and the resultant gel was weighed. For rapid viscoanalysis, a 6.7% (w/v) starch/water slurry was stirred at 960 rpm for 10 s and then at 160 rpm for the remainder of the experiment. The temperature of the sample was changed in the following manner: 50°C for 1 min, a linear increase to 95°C over 222 s, 95°C for 150 s, a linear decrease to 50°C over 228 s, and hold at 50°C for 120 s with viscosity continuously measured by an RVA4500 (Perten Instruments, Stockholm, Sweden). Starch granules were imaged by scanning electron microscopy as described in Samodien et al. [43].

For the analysis of chain lengths, isolated starch was gelatinised and debranched with isoamylase and pullulanase essentially as described previously [44]. However, prior to the analysis of the linear chains using high‐performance anion exchange chromatography with pulsed amperometric detection (HPAEC‐PAD), a dephosphorylation step was introduced to ensure all chains were measured, regardless of their phosphorylation status, using Antarctic Phosphatase (New England Biolabs), as described in [45].

## 3 Results

### 3.1 Production of Transgenic Potato Plants Repressed in SBE1 and/or GWD1

**FIGURE 1:**
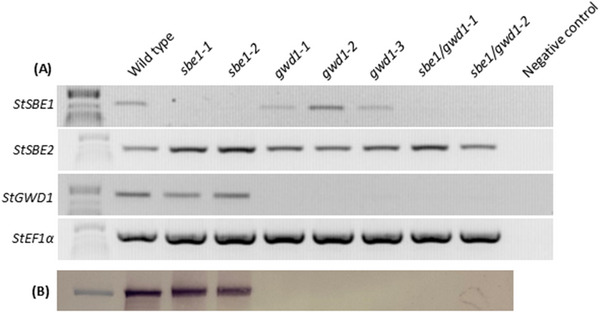
Screening of transgenic plants. (A) Semi‐quantitative RT‐PCR to examine the accumulation of *SBE1*, *SBE2*, *GWD1*, or *EF1α* (housekeeping control) transcripts in transgenic lines where either *SBE1*, *GWD1*, or both genes were simultaneously (*SBE1/GWD1*) repressed. The negative control is a sample containing no cDNA but undergoing the same PCR reaction. (B) Immunoblot examining GWD1 protein in 25 µg crude protein extracts from tubers. Full electrophoresis gels and immunoblots, as well as Coomassie stained polyacrylamide gels, are shown in Figures S1 and S2.

We used RNAi silencing constructs to repress transcript accumulation of *StSBE1* and *StGWD1* individually, or in combination with each other, and used these to generate transgenic potato lines (*sbe1‐1, sbe1‐2, gwd1‐1, gwd1‐2, gwd1‐3, sbe1/gwd1‐1*, and *sbe1/gwd1‐2)*. These were screened to demonstrate gene silencing by semi‐quantitative RT‐PCR using cDNA synthesized from tuber RNA. Transcript accumulation of *StSBE1* and *StGWD1* decreased in the transgenic lines where they were targeted (Figure 1a). Expression of *StSBE1* appeared elevated in one of the *gwd1* lines (*gwd*1*‐2*), but not the other two. *StSBE2* expression was unaffected by the RNAi constructs in most lines but was increased in both *sbe1* lines and in line *sbe1/gwd1‐1*. Immunoblots targeting GWD1 demonstrated that repression of *StGWD1* expression led to an undetectable amount of GWD1 in crude protein extracts (Figure 1b).

### 3.2 SBE1 and/or GWD1 Repression Do Not Affect Tuber Growth and Yield

**FIGURE 2:**
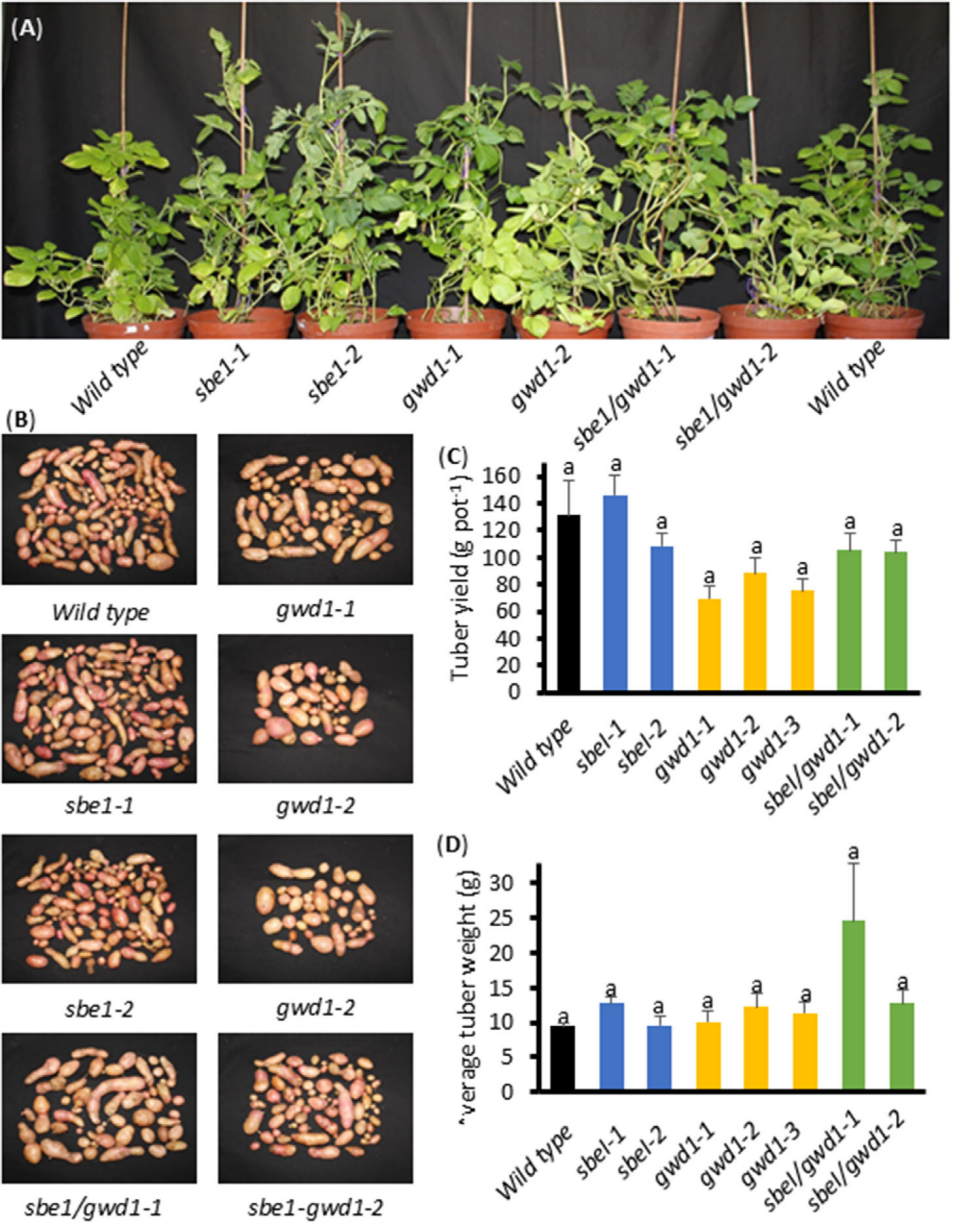
Pot trials of transgenic plants. (A) Plants after 26 weeks of growth, (B) tuber morphology, (C) tuber yield, and (D) average mass per tuber. Data represents the mean ± SEM of measurements from at least five plants. A 5% level of significance was tested using the Bonferroni–Holm post hoc test after one‐way analysis of variance.

Greenhouse‐cultivated plants were harvested after 26 weeks. The morphology of transgenic plants and harvested tubers was similar to the wild type, with minor differences in plant height observed. (Figures 2a,b). Although tuber weight per pot was reduced in the *gwd1* and *sbe1/gwd1* lines, this was not significant (Figure 2c). There were no differences in tuber number per plant (Figure 2d) or tuber starch content (Table 2) between the different lines.

**TABLE 2:** Chemical and physical characteristics of tuber starch.

Line	Apparent amylose (%) determined by Concanavalin a	Apparent amylose (%) determined by iodine binding	Tuber starch content (µmol hexose equivalents gFW^−1^)	Starch phosphate (nmol glucose 6‐phosphate µmol hexose equivalents^−1^)	Swelling power (g g^−1^)
Wild type	23.7 ± 0.6^a,b^	21.0 ± 0.4^a^	916 ± 48^a^	1.93 ± 0.05^a^	26.9 ± 1.5^a^
*sbe1‐1*	21.1 ± 0.4^b^	20.2 ± 0.9^a^	892 ± 70^a^	3.92 ± 0.02^b^	17.4 ± 0.4^b^
*sbe1‐2*	20.8 ± 1.0^b^	20.1 ± 1.0^a^	919 ± 60^a^	4.15 ± 0.02^b^	20.6 ± 1.0^a,b^
*gwd1‐1*	25.5 ± 0.4^a,c^	25.8 ± 0.3^b^	857 ± 50^a^	0.03 ± 0.01^c^	8.1 ± 0.2^c^
gwd1‐2	27.5 ± 1.0^c^	nd	nd	0.07 ± 0.02^c^	nd
*gwd1‐3*	26.1 ± 0.5^a,c^	26.2 ± 0.5^b^	920 ± 63^a^	0.17 ± 0.02^c^	10.2 ± 0.1^d^
*sbe1/gwd1‐1*	35.8 ± 0.9^d^	28.4 ± 1.0^b,c^	932 ± 55^a^	0.65 ± 0.05^d^	11.8 ± 0.1^c,d^
*sbe1‐gwd1‐2*	37.0 ± 0.4^d^	32.1 ± 1.5^c^	934 ± 44^a^	0.78 ± 0.1^d^	13.3 ± 0.1^e^

*Note*: The data represent the mean ± SEM of at least three measurements of pooled starch samples from tubers isolated from at least five plants per line. Data were analyzed by one‐way analysis of variance followed by the Bonferroni–Holm post hoc test, and letters represent groups with similar means at the 5% significance level.

Abbreviation: nd = not determined.

### 3.3 Chemical Properties of Tuber Starch

The glucose 6‐phosphate (G6P) content of tuber starch was determined enzymatically (Table 2). As reported previously [11, 12, 19, 22, 32], starch from the *sbe1* lines contained approximately double the G6P content of the starch from wild type plants, while the starch from GWD1 lines contained very little G6P. The G6P content of starch from *sbe1/gwd1* plants was greatly reduced compared to starch of the untransformed control but higher than starch of the *gwd1* lines.

Two methods were used to determine apparent amylose contents (Table 2). One was based on the binding of the lectin Concanavalin A to amylopectin, while the other relied on the differential binding of iodine to amylose and amylopectin. Both methods measured an increase in amylose in *sbe1/gwd1* lines, and the iodine binding method also indicated that amylose was increased in all *gwd1* lines. Both methodologies indicated that there was a consistent decrease in amylose in the *sbe1* lines when compared to the *gwd1* lines. Starch from all *gwd1* lines demonstrated increased amylose compared to both the wild type and *sbe1* lines, while repression of both *StSBE1* and *StGWD1* increased the amylose content significantly compared to all other lines.

**FIGURE 3:**
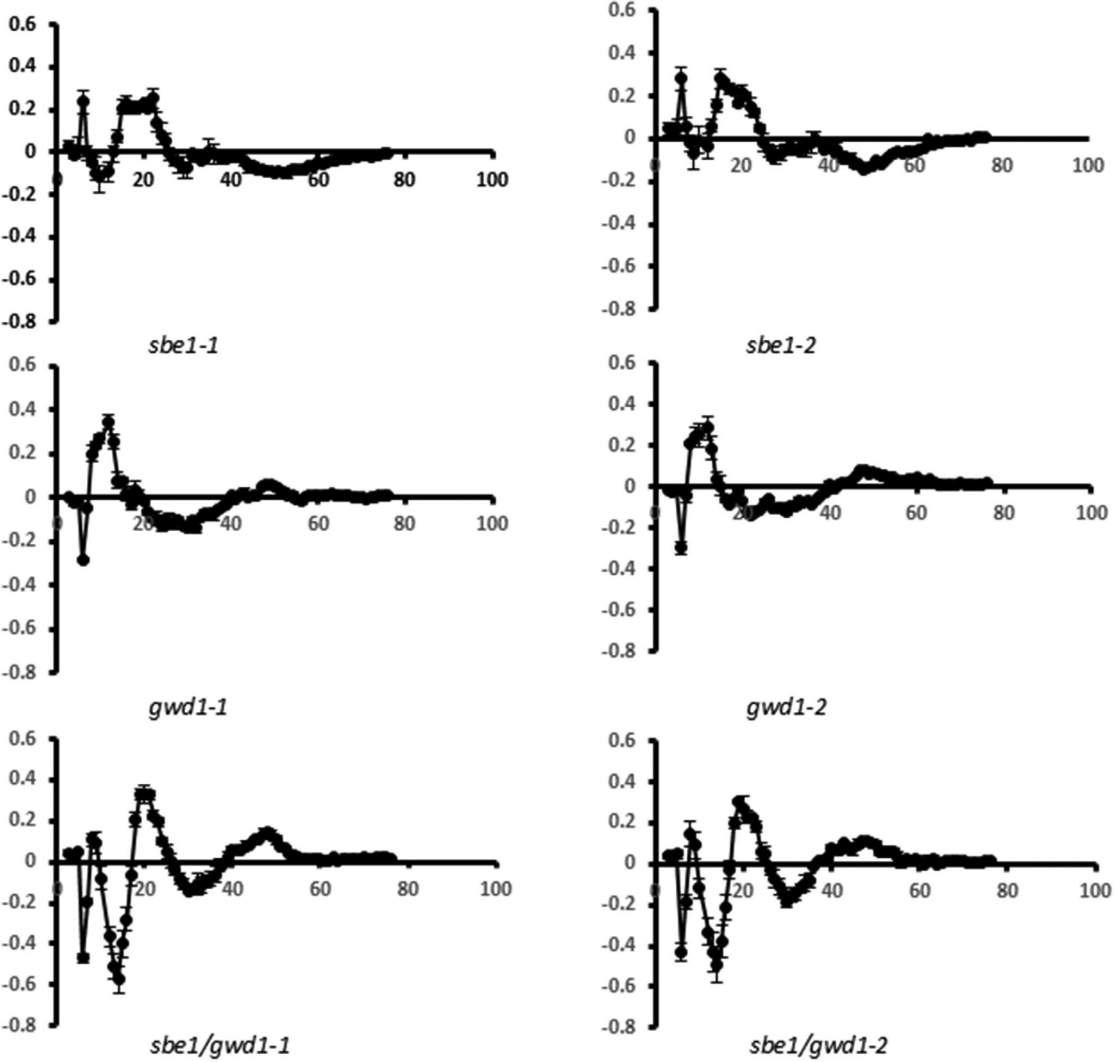
Difference plots of debranched tuber starch. Isolated starches from the lines were debranched, dephosphorylated, and separated by anion exchange chromatography (HPAEC‐PAD). Data indicate the differences between starches from the indicated transgenic lines and the wild‐type. Error bars represent SEM of three replicates. If not visible, they sit within the symbol.

Analysis of debranched and dephosphorylated starch indicated an alteration in chain length distributions of amylopectin. The starch from the *sbe1* lines contained a slightly decreased proportion of chains with DP's between DP7 and DP12 and an increased proportion between DP13 and DP21 compared to the wild‐type starch. The *gwd1*‐repressed lines contained a decreased proportion of chains of DP6 and > DP20, and an increased proportion of chains between DP8 and DP17. Starch from plants repressed in both StSBE1 and StGWD1 contained decreased proportions of chains between DP6‐7, DP11‐15, and DP27‐39 and increased proportions of chains between DP 8–9, DP17‐24, and DP42‐53 (Figure 3).

### 3.4 Starch Granule Morphology

**FIGURE 4:**
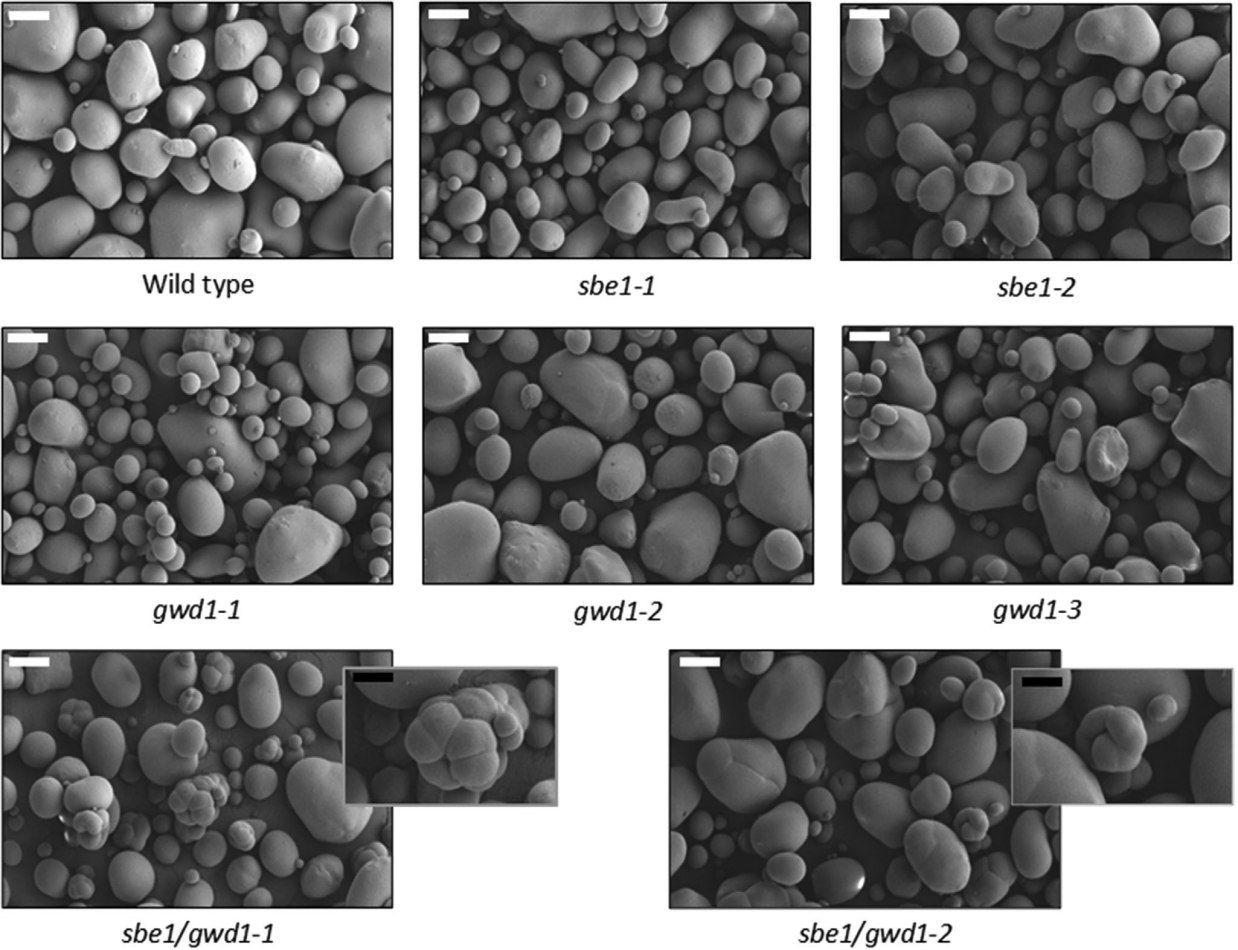
Scanning electron micrographs of purified tuber starch granules. Inset pictures show compound granules identified in the *sbe1/gwd1* lines. White scale bars represent 20 µm, and black scale bars represent 10 µm.

Scanning electron micrography of extracted starch showed that most starch granules of all lines were ovoid and smooth (Figure 4). No obvious granule morphological differences were observed in the *sbe1* or *gwd1* lines, but small numbers of multilobed granules were observed in starch isolated from both of the *sbe1/gwd1* lines.

### 3.5 Tuber Starch Physicochemical Properties

**FIGURE 5:**
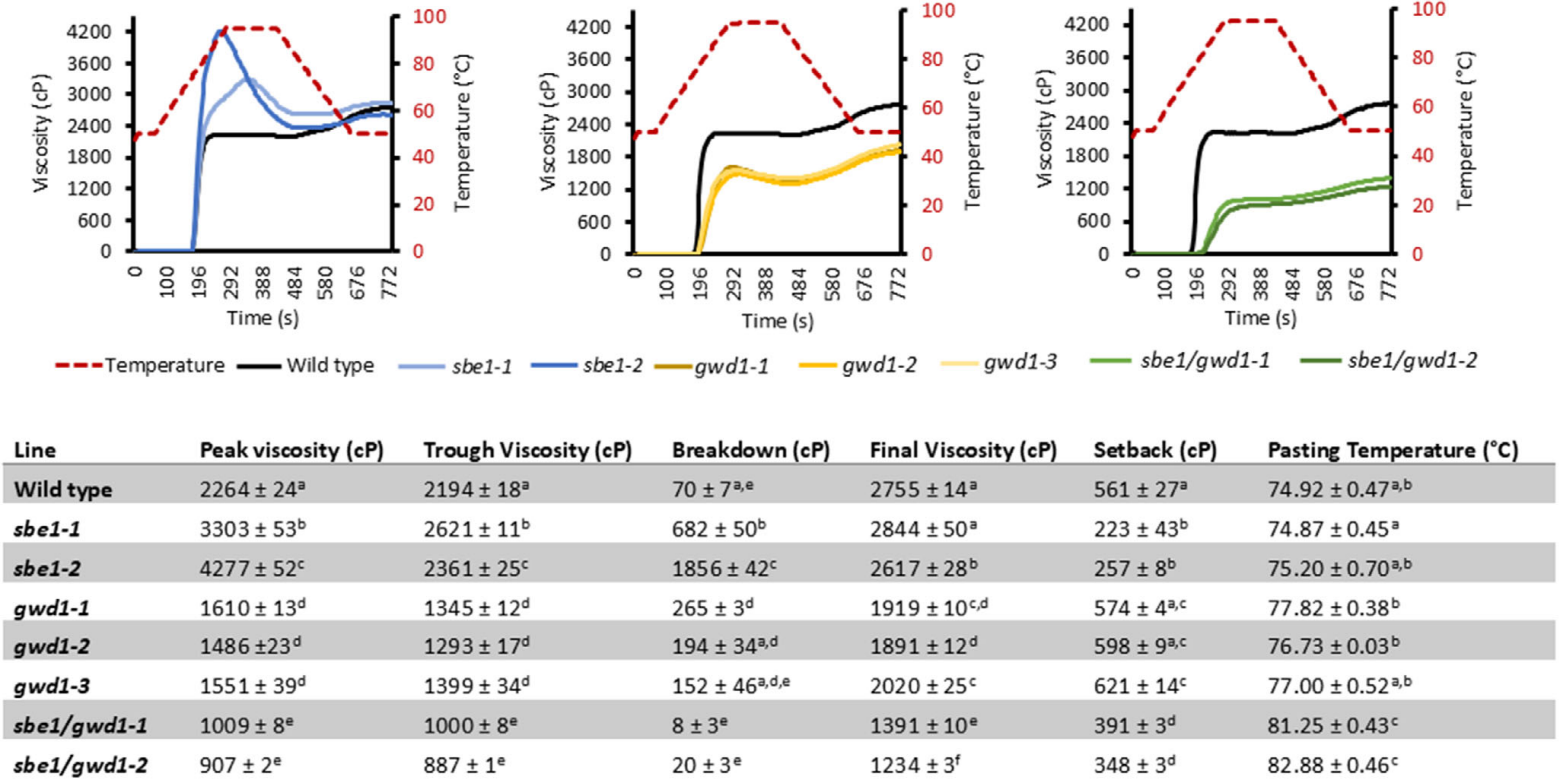
Viscosity profiles of various tuber starches measured as a starch/water paste in a rapid visco‐analyzer. Graph profiles represent the mean of three measurements, and table data represent the means ± SEM of at least three measurements for pooled starch samples isolated from tubers of at least five plants per line. Data were analyzed by one‐way analysis of variance followed by a Bonferroni post hoc test. Letters represent groups with similar means at the 5% significance level. Viscosity is measured as centipoise (cP).

Starch swelling power was assessed after heating in water, and all starches with reduced phosphate (*gwd1* and *sbe1/gwd1* lines) demonstrated reduced swelling (Table 2). Rapid viscoamylography of starch pastes was used to measure gelling properties in more detail (Figure 5). Starch from both *sbe1* lines demonstrated considerably higher peak and trough viscosities than all other lines, with starch from the *gwd1* and *sbe1/gwd1* lines showing lower values for both these measures than all other lines. Peak and trough viscosities of starch from both *sbe1/gwd1* lines were even lower than those from the *gwd1* lines. There were no consistent differences in breakdown or final viscosity, but setback was lower than the wild‐type control in all *sbe1* lines. In *gwd1* lines, the pasting temperature increased and was even higher in the *sbe1/gwd1* lines (Figure 5).

## 4 Discussion

This study focused on gaining a better understanding of how starch‐metabolizing proteins can be manipulated in potato tubers to develop starches for industrial use. Alterations in both starch branching and phosphate can affect starch physicochemical properties, leading to altered industrial usability [1]. Although the roles of StSBE1 and StGWD1 have been studied previously in transgenic or mutant potatoes where their activities were reduced or eliminated [11, 12, 18, 19, 22, 24, 27, 28, 32], no study has yet investigated how combined repression of their activities affects starch. To examine this, we manufactured transgenic potato plants repressed in expression of *StSBE1* and/or *StGWD1*.

Semi‐quantitative RT‐PCR demonstrated that our constructs reduced transcript accumulation of the target genes, but not of *StSBE2* (Figure 1). In fact, *StSBE2* expression appeared increased in both *StSBE1*‐repressed lines, and in the *sbe1/gwd1‐1* line, which is interesting as it indicates some transcriptional or post‐transcriptional upregulation of *StSBE2*. Transgenic potato plants and tubers in this study demonstrated no obvious differences in plant growth or yield, and tuber starch contents were unaltered (Figure 2; Table 2). However, further trials to assess agronomically important traits in the field would be necessary to confirm this.

The levels of covalently bound phosphate in starches isolated from the *sbe1* and *gwd1* lines, measured as glucose 6‐phosphate, were similar to those reported in the literature [11, 18, 19, 22, 24], being reduced in the *gwd1* lines and increased in the *sbe1* lines (Table 2). StGWD1 is thought to be the only enzyme responsible for phosphorylating starch at the 6‐position [46], and so any remaining starch phosphate must have been introduced by residual StGWD1. In the *sbe1/gwd1* lines, starch phosphate was lower than the untransformed control, but greater than the *gwd1* lines. This may indicate that the chimeric silencing construct containing both *StSBE1* and *StGWD1* sequences was less efficient at silencing *StGWD1* than the construct containing only the *StGWD1* sequence, even though StGWD1 protein levels appeared similarly low in all these lines (Figure 1). A more likely explanation is that because starch from the *sbe1/gwd1* lines contains more chains greater than DP40 (Figure 3), and any remaining StGWD1 would incorporate more phosphate as it demonstrates twentyfold more activity when using α‐1,4 chains that are longer than DP30 than when phosphorylating chains less than DP28 [7].

Apparent amylose was increased in the *sbe1/gwd1* lines regardless of which assay method was used and was also increased in one of the *gwd1* lines (Table 2). The increased amylose in starch from the *sbe1/gwd1* lines over that in both *sbe1* and *gwd1* lines indicates a synergistic effect of GWD1 and SBE1 repression on amylose content. Likewise, there was a synergistic effect on the amylopectin chain length distribution of starch (Figure 3). In that case, there are some similarities between the starches from these lines and those from the *sbe1* (decreased chains of between DP 8–13 and increased chains of DP 19–25) and *gwd1* (decreased chains of DP 6 and DP 23–40) lines, but also a difference to both (increased chains of DP 40–50). Such synergistic interactions on starch structure have been observed previously when starch synthases 2 and 3 were repressed simultaneously [47, 48]; the amylopectin chain length distributions of the starches from those double‐repressed lines could not be explained simply by examining the distributions of the SS2 and SS3 individual lines. In this case, we believe our data indicates that starch phosphate affects the incorporation of branch points by StSBE2. Stimulation of StSBE2 activity by starch phosphate would explain both the increased amylose detected in *StGWD1*‐repressed lines (Table 1 and refs. [12, 19]) as well as the altered constituent chains observed in starch from the *sbe1/gwd1* lines (Figure 3). In the first case, the reduced phosphate would lead to fewer branch points incorporated by StSBE2 and increased amylose, as has been demonstrated in transgenic and mutant plants where *StSBE2* expression was repressed [21, 23, 24, 27, 28]. In the case of the *sbe1/gwd1* lines, reduced incorporation of branch points by StSBE2 would occur in addition to reduced StSBE1 and lead to both increased amylose and alterations in constituent chain lengths, as has been demonstrated in lines where both *StSBE1* and *StSBE2* were repressed [23, 25, 26]. Interestingly, the lobed starch granules observed in the *sbe1/gwd1* lines (Figure 4) are similar to those found in lines where both StSBE1 and StSBE2 were repressed [26]. The formation of such granules in the *sbe1/gwd1* lines could also be explained, therefore, by an indirect reduction in SBE2 activity.

Although GWD1 has often been considered to be involved only in starch degradation, it has recently been proposed that it could also be involved in starch synthesis [16]. That study hypothesized that phosphate incorporation by GWD1 could affect granule architecture by facilitating the activity of starch biosynthetic enzymes. Although starch phosphate is not known to promote the activity of SBE, it has been demonstrated in Arabidopsis leaves to increase the ability of β‐amylase to degrade starch [51, 52] and to decrease the ability of an isoform of starch synthase to elongate glucan chains [53]. This is most likely through the phosphate disrupting double helical glucan chains within amylopectin, making them more accessible [4, 51, 54], and we hypothesize that such a structure is important for StSBE2 activity. A recent study has shown that branching enzymes from a variety of prokaryotes and eukaryotes demonstrate increased affinity to α‐1,4‐polyglucans lacking covalently bound phosphate than to ones with phosphate [33]. This would imply that the reduced starch phosphate in the *gwd1* and *sbe1/gwd1* lines should lead to increased branching by StSBE2 and not the decreased amounts observed. However, although that study included StSBE1, it did not include StSBE2, and so it is possible that the two enzymes show opposite affinities to these polyglucans. If our hypothesis is incorrect, then the alterations in starch structure must be caused by an alteration in the activity of other enzymes. This may be due to alterations in starch‐bound phosphate affecting the activity of other starch biosynthetic enzymes, or disruption of enzyme complexes involved in starch synthesis, which are known to occur in some plants [49, 50].

Various physical properties of starch are influenced by the phosphate content, such as swelling power (which indicates the granules ability to hold water without bursting) and gelatinization [55, 56, 57, 58]. We measured swelling both directly (Table 2) and indirectly by examining pasting temperature and peak viscosity (Figure 5), both of which are directly influenced by swelling power [59]. As previously reported [60], all of these parameters were reduced in the GWD1 lines, but we observed that they were also reduced in the *sbe1/gwd1* lines. Both amylose content and increased length of constituent chains are negatively correlated with swelling power [61, 62, 63], while phosphate content is positively correlated [43, 64] with that parameter. The increased amounts of amylose, increased constituent chain lengths, and decreased starch phosphate found in starch from the *gwd1* and *sbe1/gwd1* lines would, therefore, help to explain their decreased swelling power. Interestingly, we found no alteration in swelling power in the *sbe1* lines, despite the increased starch phosphate (Table 2) and the increased peak viscosity (Figure 5). This suggests that another structural alteration may counteract the influence of the increased phosphate in those lines, most likely the increased lengths of constituent chains observed in their starch (Figure 3). Pasting properties of starch from the *sbe1/gwd1* plants were clearly altered compared to the *gwd1* or *sbe1* lines. Peak, trough, and final viscosities, as well as breakdown and setback, were all reduced compared to starch from all other lines. This is likely caused by the significantly increased amylose content in these lines and the increased constituent chain lengths, both of which are thought to reduce peak viscosity [63].

The pasting properties of these starches suggest potential industrial uses. Potato starch is often used in the food industry because it forms a clear paste and has both a neutral flavor and relatively high swelling power. This has led it to be used in products such as noodles [65]. However, the low viscosity demonstrated by the SBE1/GWD1 lines makes them more suitable to be used in batters [66]. Similarly, the high phosphate present in potato starch means that it is useful in the paper industry because of the charge it carries. Although the phosphate content of starch from the SBE1/GWD1 lines is lower than the control, it is still greater than that found in many cereal starches [67], and the presence of phosphate alongside the lower viscosity could make it useful as a coating for some types of paper.

Taken together, this study highlights that the interplay between genetic modifications leads to alterations of starch characteristics and physicochemical properties. The traits examined here are influenced by the cumulative effects of alterations in starch branching structure and phosphate content when *StSBE1* and/or *StGWD1* are repressed. This has led to starches in the *sbe1/gwd1* lines that are altered in chemical and physicochemical properties in ways that could not be predicted based on examination of the individual *StSBE1* and *StGWD1* repressed lines. We believe that to fully understand how starch biosynthetic enzymes affect starch structure, examinations of functional interactions between many combinations of enzymes involved in starch biosynthesis need to be made.

## Author Contributions

Jens Kossman and James R. Lloyd designed the research. Pedri Claassens made the silencing constructs, which were transformed into potato by Muyiwa Adegbaju with advice from Christell van der Vyver. Analysis of transgenic plants was performed by Muyiwa Adegbaju and Nina Gouws, except for the analysis of constituent chain lengths, which was performed by Michaela Fischer‐Stettler and Samuel C. Zeeman. All authors except Jens Kossmann were involved in the writing of the manuscript. The authors would like to thank Prof Marena Manley and Dr Stephan Hayward for their help with the RVA analysis.

## Conflicts of Interest

The authors declare no conflicts of interest.

## Supporting information




**Supporting file 1**: biot70051‐sup‐0001‐FiguresS1‐S2.docx.

## Data Availability

The data that support the findings of this study are available from the corresponding author upon reasonable request.
