# Supplementary material for: Simultaneous Repression of GLUCAN WATER DIKINASE 1 and STARCH BRANCHING ENZYME 1 in Potato Tubers Leads to Starch With Increased Amylose and Novel Industrial Properties
Source: Biotechnol J. 2025 Jun 9;20(6):e70051. doi: 10.1002/biot.70051 (PMC12149484; doi:10.1002/biot.70051)
Supplement: Supplementary file 1 — Supporting file 1: biot70051‐sup‐0001‐FiguresS1‐S2.docx. [file BIOT-20-e70051-s001.docx]

**Supplementary Figures**

**Supplementary Figure S1** Uncropped gel images of semi-quantitative RT-PCR to examine accumulation of *SBE1*, *SBE2*, *GWD1* or *EF1α* (housekeeping control) transcripts in transgenic lines where either *SBE1*, *GWD1*, or both genes simultaneously (*SBE1/GWD1*), were repressed. The negative control is a sample containing no cDNA but undergoing the same PCR reaction.

**Supplementary Figure S2** Uncropped gel images of immunoblot examining GWD1 protein in 25 µg crude protein extracts from tubers and Coomassie stained gel representing loading of protein..

**
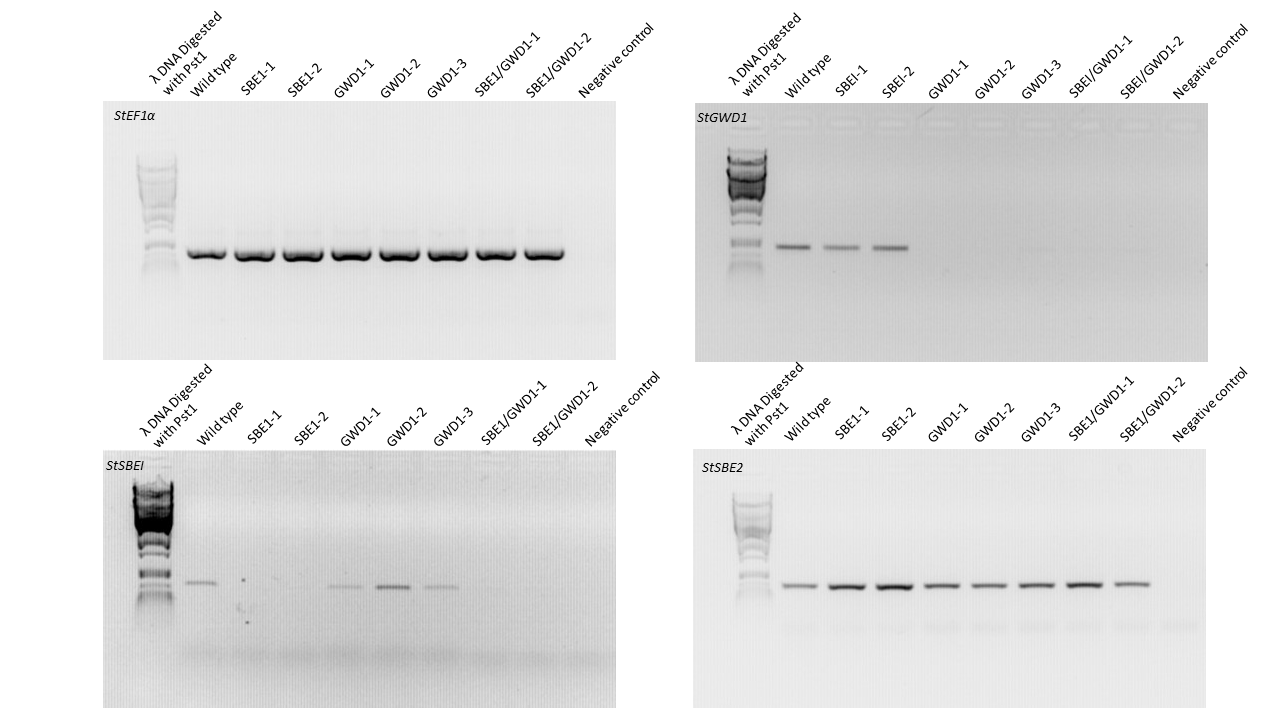
**

**Supplementary Figure S1**

**
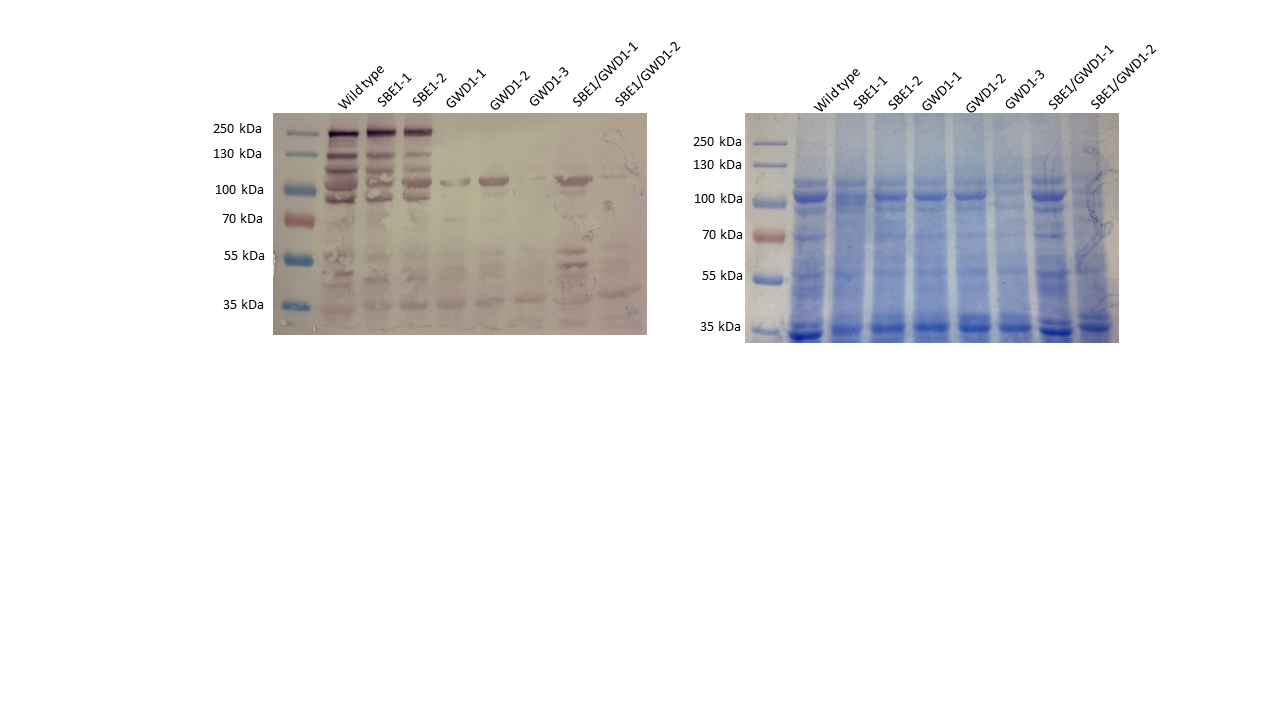
**

**Supplementary Figure S2**
